# Mechanistic Modeling of Mycobacterium tuberculosis Infection in Murine Models for Drug and Vaccine Efficacy Studies

**DOI:** 10.1128/AAC.01727-19

**Published:** 2020-02-21

**Authors:** Nan Zhang, Natasha Strydom, Sandeep Tyagi, Heena Soni, Rokeya Tasneen, Eric L. Nuermberger, Rada M. Savic

**Affiliations:** a University of California San Francisco, San Francisco, California, USA; b Center for Tuberculosis Research, Department of Medicine, Johns Hopkins University School of Medicine, Baltimore, Maryland, USA

**Keywords:** *Mycobacterium tuberculosis*, TB disease, mouse model, mechanistic modeling, adaptive immunity, drug discovery and development, vaccine, pyrazinamide

## Abstract

Tuberculosis (TB) drug, regimen, and vaccine development rely heavily on preclinical animal experiments, and quantification of bacterial and immune response dynamics is essential for understanding drug and vaccine efficacy. A mechanism-based model was built to describe Mycobacterium tuberculosis H37Rv infection over time in BALB/c and athymic nude mice, which consisted of bacterial replication, bacterial death, and adaptive immune effects. The adaptive immune effect was best described by a sigmoidal function on both bacterial load and incubation time. Applications to demonstrate the utility of this baseline model showed (i) the important influence of the adaptive immune response on pyrazinamide (PZA) drug efficacy, (ii) a persistent adaptive immune effect in mice relapsing after chemotherapy cessation, and (iii) the protective effect of vaccines after M. tuberculosis challenge. These findings demonstrate the utility of our model for describing M. tuberculosis infection and corresponding adaptive immune dynamics for evaluating the efficacy of TB drugs, regimens, and vaccines.

## Abstract (precis)

Tuberculosis (TB) drug, regimen, and vaccine development rely heavily on preclinical animal experiments, and quantification of bacterial and immune response dynamics is essential for understanding drug and vaccine efficacy. A mechanism-based model was built to describe Mycobacterium tuberculosis H37Rv infection over time in BALB/c and athymic nude mice, which consisted of bacterial replication, bacterial death, and adaptive immune effects.

## INTRODUCTION

Tuberculosis (TB) caused by Mycobacterium tuberculosis infection has existed for thousands of years, and it is currently one of the top ten causes of death worldwide. In 2017, an estimated 1.3 million HIV-negative people and 0.3 million HIV-positive people died from TB, 10.0 million people fell ill with TB, and more than half a million had multidrug-resistant TB (MDR-TB) (1). After several decades of inactivity, substantial efforts have been made in the last 2 decades to develop new TB drugs, regimens, and vaccines capable of ending the TB pandemic (2, 3).

Infection with M. tuberculosis starts with inhalation of airborne droplet nuclei containing viable bacilli. The infected phagocytic cells in the lung, mainly macrophages, will recruit additional macrophages and other immune cells, such as neutrophils, monocytes, and dendritic cells (DCs), from neighboring blood vessels. As these cells are recruited, they become infected by the expanding population of mycobacteria and establish early granulomas, which are a pathological hallmark of TB (4–6). The immune response to M. tuberculosis develops slowly. The early innate immune response has little immediate antimycobacterial effect (5, 6). As a result, bacterial spread and replication are still uninterrupted during this stage (5, 6). The increasing bacterial population expands the infection range to include DCs, which subsequently migrate to the draining lymph nodes of the lung. The antigens presented by DCs then activate the naive antigen-specific T cells, initiating an adaptive immune response. The adaptive immune response suppresses the replication of bacteria (6).

Effective adaptive cell-mediated immune responses to M. tuberculosis are critical to controlling TB, as demonstrated by the adverse impacts of primary and acquired cell-mediated immune deficiency and the beneficial effects of priming and boosting cell-mediated immune responses by Mycobacterium bovis bacillus Calmette-Guérin (BCG) vaccination (7). Adaptive immune responses also improve the efficacy of chemotherapy for TB. Combination chemotherapy results in lower rates of bacterial elimination and higher relapse rates in athymic nude mice compared to those in immunocompetent BALB/c mice (8–10). The antibacterial effect of the first-line drug pyrazinamide (PZA) may be particularly dependent on cell-mediated immune responses, as it is strongly correlated with the local pH and probably exerts its maximal sterilizing effects in the acidified microenvironment of phagosomes, where the pH can be as low as 4.5 after macrophage activation by antigen-specific helper T cells (11, 12). Therefore, the role of the adaptive immune response to M. tuberculosis must be accounted for in the development of novel preventive and therapeutic strategies for TB disease.

Animal models of TB are widely used for developing TB drugs, vaccines, and diagnostic reagents, and for research on TB pathogenesis and drug target identification (13). A number of animal species have been used for TB models, including mice, guinea pigs, rabbits, and nonhuman primates. Each model has its pros and cons, including the extent to which it represents human TB with respect to clinical signs, pathological changes, bacterial loads in organs, disease progression, and immunological parameters (13, 14). Among these animal species, mouse models have demonstrated predictive value for evaluating the efficacy of drug regimens in clinical use (15). Specifically, BALB/c and athymic nude mice have been used as immunocompetent and immune-deficient TB models, respectively, in preclinical efficacy studies. Assuming infection with a virulent strain of M. tuberculosis, the infectious dose, the incubation time, and the onset and efficacy of the adaptive immune response primarily determine whether infected mice will limit bacterial replication and contain the infection or succumb to an overwhelming infection (16). The type of model employed can therefore have a profound impact on the interpretation of drug or vaccine effects (15, 17). As such, an appropriate understanding of the relationships between the infection conditions and the development of effective adaptive immune responses is critical in applying murine TB models appropriately in research.

Despite the important role of the adaptive immune response in suppressing bacterial replication and facilitating the action of TB drugs, quantitation of adaptive immune effects has been challenging due to the complexity of the immune response. Various mathematical models have been developed to describe immunological responses to pathogens at the molecular, cellular, and tissue levels (18–25). We recently developed a mathematical model describing bacterial growth, an adaptive immune effect, and drug treatment effects, established a precedent for using a baseline model of M. tuberculosis infection in mice for determining pharmacokinetics/pharmacodynamics (PK/PD) relationships for TB drugs, and translated the findings into human trial simulations to predict treatment outcomes (26). However, the baseline model developed in that work described the adaptive immune effect as a function of incubation time relying on the difference in lung CFU counts observed between immunocompetent BALB/c mice and immune-deficient athymic nude mice, which did not account for the limited adaptive immune response that occurs in athymic nude mice due to T-cell maturation outside the thymus (27). In addition, this approach did not explicitly account for the influence of bacterial number on the development of the adaptive immune effect in BALB/c mice, especially when CFU counts in different mice happen to be the same, although the size of the infectious dose and the incubation time are different. Because diverse mouse models, including acute, subacute, and chronic infection models, are used in TB drug development to understand antibacterial activity under time-varying immunological conditions against bacterial populations of differing sizes and growth rates, both during and after treatment, wider application of a baseline infection model requires consideration of these factors.

In the current work, we integrate the impact of both bacterial burden over time without treatment and infection incubation time and develop a general mechanism-based baseline model of the bacterial infection, reinfection, and relapse profile and underlying adaptive immune system response. To that end, we pool data from 86 experiments in BALB/c and nude mice of various infection models (acute, subacute, and chronic) with various incubation periods and infection loads. Our objective is to demonstrate that this baseline model can be applied for numerous scenarios of preclinical studies to quantitatively describe and understand the involvement of adaptive immune responses and the corresponding effects of various treatment interventions.

## RESULTS

### Differential bacterial growth profile in BALB/c mice and athymic nude mice.

**FIG 1:**
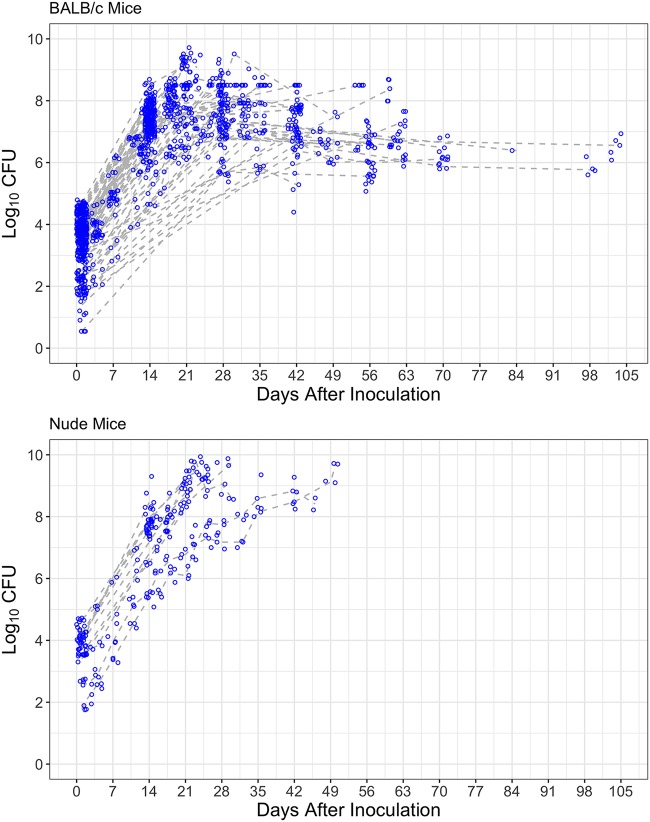
Raw data of bacterial growth in BALB/c mice (top) and athymic nude mice (bottom). Circles indicate raw data observed; dashed lines indicate median values of raw data.

In total, 74 and 12 studies evaluating various drugs, drug combinations, and infectious dose-response effects in the absence of treatment after aerosol infection of BALB/c and athymic nude mice, respectively, were collected. The data from the untreated control arm of each study were pooled for modeling the bacterial burden dynamics in the lungs over time. A spaghetti plot shows the bacterial growth profiles in each mouse strain (Fig. 1). As expected, the size of the infectious dose determined the peak CFU count in BALB/c mice, and higher infectious doses (e.g., >3 log_10_ CFU) caused mouse death more often than did lower infectious doses after the same incubation period, e.g., 28 days, in both mouse strains (Fig. S1).

### Model describing bacterial growth dynamics in BALB/c and athymic nude mice.

The most parsimonious mechanism-based model to describe mycobacterial growth was adopted, which contained a single bacterial compartment with first-order rate constants for bacterial replication (*K_g_*) and bacterial death (*K_d_*) (28). Despite being initiated upon the inhalation of infectious aerosol droplets, the innate immune response has little immediate antimycobacterial effect, as mentioned previously (6). The onset of the more effective adaptive cell-mediated immune response is delayed until 11 to 14 days after inoculation in BALB/c and other immunocompetent mice (5, 6). Therefore, our baseline model included parameters for bacterial replication, bacterial natural death, and bacterial growth control by the adaptive immune response in the mouse lung (16, 28). Because the increase in bacterial number exhibited a log-linear profile up to 2 weeks after inoculation, the change in bacterial number (*B*) over time was informed by the difference of the bacterial replication rate (*K_g_*) and the natural death rate (*K_d_*), with minimal contribution of the inhibitory adaptive immune effect during the early incubation stage. Under those conditions, the initial growth dynamics of the bacteria can be explained by equation 1. Since only the result of the net growth rate (*K_g_* − *K_d_*) can be estimated, *K_d_* in the model was fixed to a literature value (0.41 day^−1^) (29), and *K_g_* was then estimated.

(1) $$\frac{\mathrm{dB}}{\mathrm{dt}}=K_{g}\times B-K_{d}\times B$$

To supplement the preexisting study data that we collected, we performed an additional experiment that investigated the impact of the infectious dose size on bacterial growth in BALB/c and athymic nude mice with intensive sampling time points for CFU counts (Fig. S2). The intensive sampling in that experiment revealed an initial lag period after inoculation in which bacterial growth was extremely slow. Therefore, a turning point was used to differentiate the slow growth right after inoculation and the rapid growth developing afterwards. As such, for all BALB/c studies, *K_g_* was estimated as two different values during the early incubation period. Based on the observed data, day 4 postinfection was identified as the turning point at which the *K_g_* value changed to reflect rapid bacterial replication.

The timing of the onset of the adaptive immune effect was then added. The observed data in BALB/c mice and estimated *K_g_* value revealed that the bacterial growth rate decreased slightly as the length of incubation increased from 7 days to 18 days postinfection across studies before reaching a plateau, which implied the slow onset of an effective adaptive immune response over the first 3 weeks of incubation. In contrast, although bacterial replication in athymic nude mice after low infectious doses started slowing at a similar time, replication continued until the end of the incubation period or the death of the mice. This confirms that the adaptive immune response is significantly less effective in athymic nude mice compared to that in BALB/c mice. With high infectious doses of more than 3 log_10_ CFU, BALB/c mice and athymic nude mice both would die shortly after the early stage due to the high burden of bacteria in the lung and the insufficient time for the immune effect to respond.

**FIG 2:**
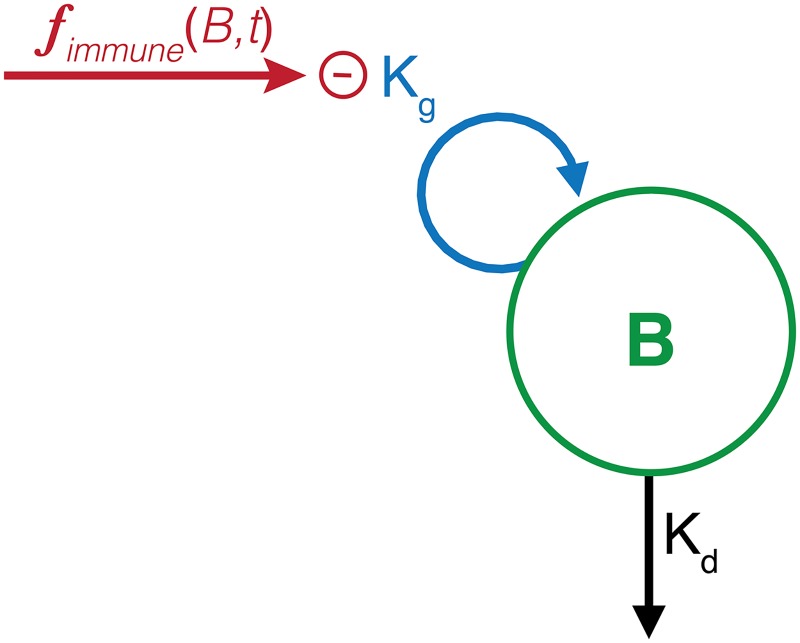
Structural baseline model. A single bacterial compartment (*B*) with first-order rate constants for logarithmic bacterial replication (*K_g_*) and natural bacterial death (*K_d_*) was used to describe the rapid growth of bacteria during the early infection phase. The adaptive immune effect that controls bacterial infection was described by the following sigmoidal function on both bacterial load and incubation time: $f_{\mathrm{immune}}^{}(B,t)$. The bacterial growth rate is decreasing as the development of the adaptive immune effect depending on the bacterial number and time of incubation.

The adaptive immune effect in BALB/c mice was included in the model using data from both early and late incubation stages. The adaptive immune effect on the bacterial replication was previously described as a nonlinear (sigmoidal) function of bacterial load, with maximal immune effect (*K_B_*) and bacterial load that stimulates half of the maximal effect (*B*_50_) (28, 30–32). A Hill coefficient (γ*_B_*) with an initial value of 1 was added to allow for the estimation of the steepness of the sigmoidal curve. As such, the change in bacterial number over time was made dependent on the bacterial replication rate (*K_g_*) using a first-order function with the adaptive immune effect using a sigmoidal function as an inhibitory effect on bacterial growth ($1-[(K_{B}\times B^{\gamma_{B}})/(B_{50}^{\gamma_{B}}+B^{\gamma_{B}})]$) (equation 2) (Fig. 2).

(2) $$\frac{\mathrm{dB}}{\mathrm{dt}}=K_{g}\times B\times (1-\frac{K_{B}\times B^{\gamma_{B}}}{B_{50}^{\gamma_{B}}+B^{\gamma_{B}}})-K_{d}\times B$$

**FIG 3:**
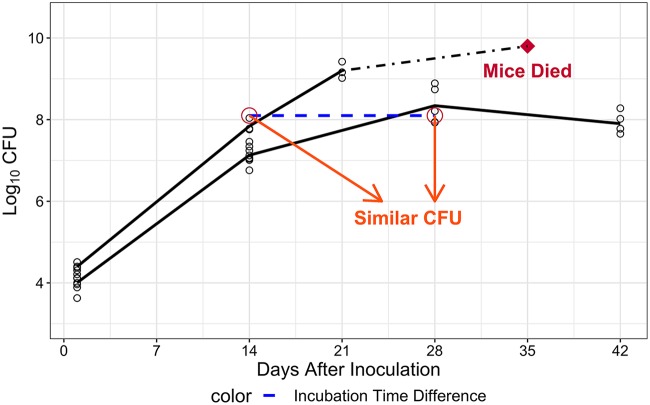
Impact of incubation time on bacterial infection in BALB/c mice. Although inoculum size differed by only half a log, bacterial infection showed nonparallel profiles and different infection outcomes in mice. The CFU count for the dead mice is an estimate based on experience.

The timing of the onset of adaptive immunity is critical to the dynamics of bacterial infection. The delayed onset of the adaptive immune response after primary infection allows bacteria to multiply up to a point at which protective adaptive immunity is activated to suppress bacterial multiplication and control the infection. The size of the infectious dose plays an important role in the ability of the infected host to control the infection, namely, the higher the infectious dose, the sooner the bacterial number approaches a tipping point where it exceeds the ability of the host to survive the infection even if treatment is initiated. Lower, nonlethal infectious doses allow sufficient time for the adaptive immune effect to constrain bacterial multiplication before reaching this tipping point. However, the level at which the bacterial burden peaks prior to containment is directly correlated with the size of the infectious dose, as is the subsequent plateau at which the bacterial burden stabilizes, which may be lower than the peak if the adaptive immune effect is strong enough (16). In our studies, it was evident that mice receiving high infectious doses died with high (and increasing) bacterial burdens at the later incubation stage (i.e., 3 to 5 weeks postinfection), although the bacterial numbers in those mice on day 14 postinfection were often similar to the bacterial numbers on day 28 postinfection in mice receiving lower infectious doses that prevented further multiplication, which did not die (Fig. 3). Therefore, bacterial number is not the only variable that determines the magnitude of the adaptive immune effect in that different incubation times could result in the same CFU counts in the lungs after different infectious doses, where the adaptive immune effect could be significantly different. As such, a time-dependent adaptive immune effect component (time covariate) was formulated as a sigmoidal function [$\left(K_{T}\times t^{\gamma_{T}})/(T_{50}{}^{\gamma_{T}}+t^{\gamma_{T}}\right)$], with *T*_50_ as the number of days required to reach half of the time covariate effect, *K_T_* as the maximal magnitude of time covariate effect, and γ*_T_* as the steepness of the time covariate curve versus time. The time covariate was added simultaneously with the CFU-dependent adaptive immune effect component [$\left(K_{B}\times B^{\gamma_{B}})/(B_{50}^{\gamma_{B}}+B^{\gamma_{B}}\right)$], as an inhibitory effect on bacterial growth [$\left(1-[(K_{B}\times B^{\gamma_{B}})/(B_{50}^{\gamma_{B}}+B^{\gamma_{B}})]\right)\times \left(1-[(K_{T}\times t^{\gamma_{T}})/(T_{50}{}^{\gamma_{T}}+t^{\gamma_{T}})]\right)$] (equation 3). This reformulated baseline model better described the bacterial burden over time in BALB/c mice compared to the previous model with the adaptive immune effect solely dependent on bacterial number, regardless of whether the immune effect function was implemented on the bacterial growth or death component (change of objective function value [ΔOFV] = −944.407 and −819.73, respectively; degrees of freedom [df] = 4; *P* < 0.01). The inhibitory effect on bacterial growth was superior to the stimulatory effect on bacterial death (ΔOFV = 45.498, df = 0; *P* < 0.01).

(3) $$\frac{\mathrm{dB}}{\mathrm{dt}}=K_{g}\times B\times (1-\frac{K_{B}\times B^{\gamma_{B}}}{B_{50}^{\gamma_{B}}+B^{\gamma_{B}}})\times (1-\frac{K_{T}\times t^{\gamma_{T}}}{T_{50}{}^{\gamma_{T}}+t^{\gamma_{T}}})-K_{d}\times B$$

It is known that immune-deficient athymic nude mice have a modest amount of T-cell maturation outside the thymus, so they do not have a completely absent adaptive immune response (27). As a result, a similar baseline model was built for athymic nude mice, in which change in bacterial number over time was dependent on bacterial replication rate and natural death rate in a first-order function, and the adaptive immune effect was a sigmoidal function of bacterial load and time individually added on bacterial growth rate (equation 3). This reformulated baseline model provided a better fit to the data than the preceding model without a time covariate, even for the minimal adaptive immune effect in athymic nude mice (ΔOFV = −128.205; df = 3; *P* < 0.01). The goodness of fit plots show that the structural and residual error models are able to describe the data of BALB/c and athymic nude mice well (Fig. S3A and B, respectively).

**FIG 4:**
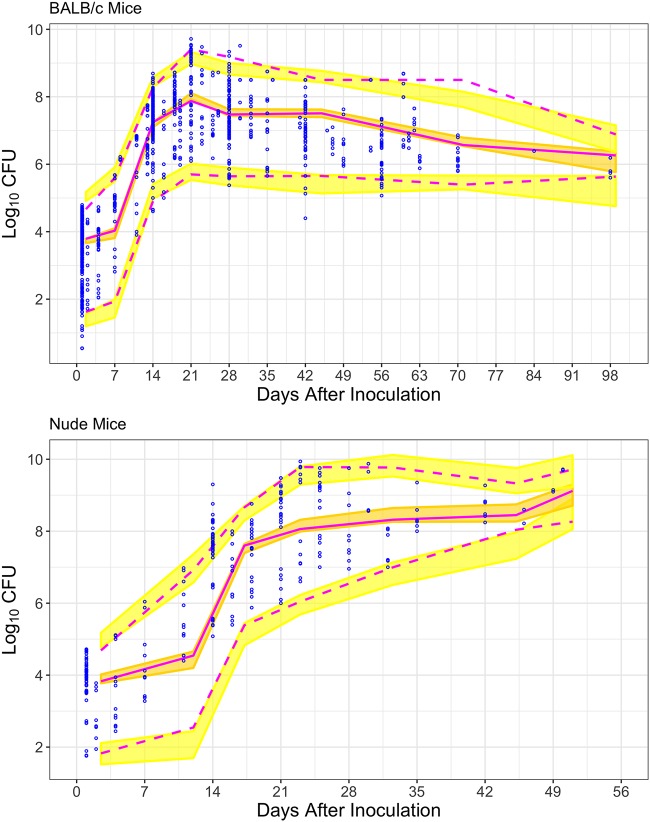
Visual predictive checks for baseline model of BALB/c mice (top) and athymic nude mice (bottom) (*N* = 200). The final baseline model for both BALB/c and athymic nude mice is able to describe the observed data.

Visual predictive check (VPC) inspections indicated that for both mouse models, the median and 95% confidence interval (CI) of the observed data were consistently within the 90% CI of the median and the 95% prediction interval of the simulated bacterial number (Fig. 4).

**FIG 5:**
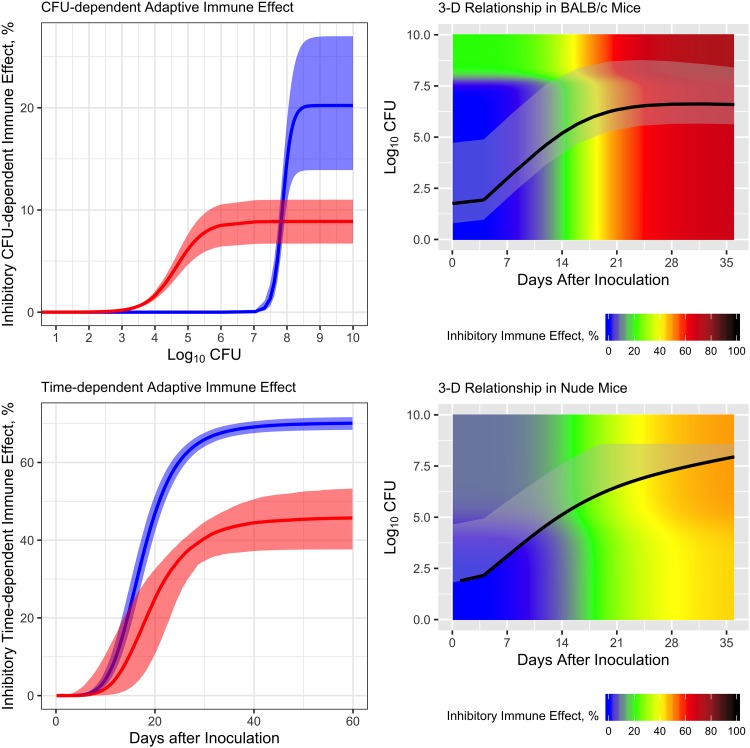
Model behavior. (A) Relationship between bacterial number and CFU-dependent adaptive immune effect (top left) and relationship between incubation days and time-dependent adaptive immune effect (bottom left) in BALB/c mice and athymic nude mice. Shaded area indicates the uncertainty of parameter estimates. (B) Relationship between bacterial number, incubation days, and total adaptive immune effect in BALB/c mice (top right) and athymic nude mice (bottom right). Black line shows a typical profile observed; gray shaded area is the simulated range of bacterial number throughout incubation using the inoculums of the observed data.

Overall, the adaptive immune effect on bacterial replication is dependent on the bacterial number and incubation time. The maximal level of the adaptive immune effect stimulated in BALB/c mice is much higher than that in athymic nude mice (Fig. 5). Since there is minimal adaptive immune response in athymic nude mice, the estimate of maximal adaptive immune effect that comes from the bacterial number (*K_B_*) for athymic nude mice model is lower than that for BALB/c mice (9.01% versus 20.3%) and requires a lower bacterial number (*B*_50_) to reach half of that level (4.65 versus 7.86 log_10_ CFU) (Table 1 and Fig. 5, top left). The maximal adaptive immune effect that comes from the incubation time (*K_T_*) for athymic nude mice model is also lower than that for BALB/c mice (46.2% versus 70.2%). Interestingly, the time to reach half of the maximal time covariate was slightly longer in athymic nude mice than in BALB/c mice (19.2 versus 17.4 days) (Table 1 and Fig. 5, bottom left). In both mouse models, the time-dependent effect component dominates the development of the adaptive immune effect with a higher *K_T_* over *K_B_* (Table 1).

**TABLE 1:** Parameter estimates of baseline model for BALB/c and athymic nude mice

Parameter (unit)	Description	Values (relative SD [%]) for:	
		BALB/c mice	Athymic nude mice
*K_g_* (day^−1^)	Bacterial growth rate	1.22 (3)	1.14 (5)
*K_d_* (day^−1^)	Bacterial death rate	0.41 (fixed)	0.41 (fixed)
*K_B_* (%)	Maximal inhibitory CFU-dependent adaptive immune effect	20.3 (19)	9.01 (15)
*B*_50_ (log_10_ CFU)	CFU counts to reach half of *K_B_*	7.86 (3)	4.65 (13)
γ*_B_*	Steepness of the CFU-dependent adaptive immune effect curve	3.11 (10)	
*K_T_* (%)	Maximal inhibitory time-dependent adaptive immune effect	70.2 (1)	46.2 (11)
γ*_T_*	Steepness of the time-dependent adaptive immune effect curve	5 (5)	4.78 (28)
*T*_50_ (days)	Time to reach half of maximal time covariate	17.4 (4)	19.2 (15)

### Bacterial relapse and preimmunity.

We previously observed that the bacterial burden in the lungs of BALB/c mice relapsing after combination chemotherapy increases with time after treatment completion but peaks around 4 log_10_ CFU, a level significantly lower than that observed with primary infection. Therefore, we presumed that the adaptive immune response reacts differently (and more effectively) during the relapse due to the immunological memory from the primary infection. It is known that, in the first 2 to 3 weeks after primary infection, the bacteria multiply in the lung under the negligible antibacterial effect of the predominantly innate immune response and disseminate from the lung via the lymphatic drainage (5, 6, 16). Once the bacteria arrive in the draining lymph nodes, T cells are slowly activated within the draining lymph node to initiate acquired cell-mediated immunity, and antigen-specific memory is triggered at the same time. Then, the acquired cellular immunity is initiated (16, 33). In order to explore the dynamics of the adaptive immune effect during and after combination chemotherapy, the combined drug effect (EFF) of a first-line regimen of rifampin-isoniazid-pyrazinamide (RHZ) was first estimated using historical data with the assumption that the adaptive immune effect remains at its peak, which is reached close to the time when drug treatment started (day 32 postinfection), until the end of treatment (day 102 postinfection). First, in simulations, our baseline model was able to predict the CFU counts before treatment started. For RHZ treatment beginning 32 days postinfection, the drug effect (EFF) was assumed to be constant during each period between sampling time points and estimated as either a percent inhibition of bacterial growth or a percent acceleration of bacterial death, which turned out to have the same OFV values (equations 4 and 5). To simplify the interpretation, the model with the drug effect (EFF) as an inhibitory effect on bacterial growth was used. The effect estimate of RHZ was 71.9% inhibition for days 0 to 14 of treatment, 52.5% inhibition for days 14 to 28 of treatment, and 33.4% inhibition for days 28 to 70 of treatment using our model.

(4) $$\frac{\mathrm{dB}}{\mathrm{dt}}=K_{g}\times B\times (1-\frac{K_{B}\times B^{\gamma_{B}}}{B_{50}^{\gamma_{B}}+B^{\gamma_{B}}})\times (1-\frac{K_{T}\times t^{\gamma_{T}}}{T_{50}{}^{\gamma_{T}}+t^{\gamma_{T}}})\times (1-\text{EFF})-K_{d}\times B$$

(5) $$\frac{\mathrm{dB}}{\mathrm{dt}}=K_{g}\times B\times (1-\frac{K_{B}\times B^{\gamma_{B}}}{B_{50}^{\gamma_{B}}+B^{\gamma_{B}}})\times (1-\frac{K_{T}\times t^{\gamma_{T}}}{T_{50}{}^{\gamma_{T}}+t^{\gamma_{T}}})-K_{d}\times B\times (1+\text{EFF})$$

**FIG 6:**
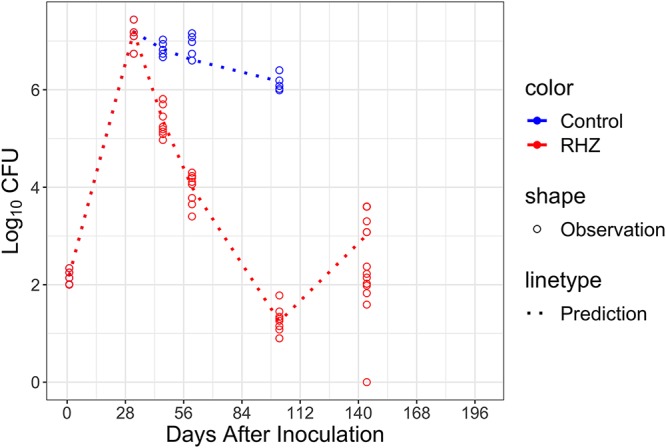
Prediction of bacterial relapse in BALB/c mice after RHZ treatment. Using a shorter *T*_50_, our baseline model is able to predict the bacterial number during the relapse.

Having observed that bacteria started regrowing after removal of the antibacterial effect of the drug treatment, we presumed that immune recall responses were likely initiated following a similar but much faster process due to the memory of antigen-specific T cells (16, 33). It is even possible that the adaptive immune effect never disappeared entirely, since there were always live bacteria and bacterial antigens in the lung. As such, we extended our model to describe bacterial regrowth during relapse by adopting an accelerated adaptive immune response. We first reestimated *T*_50_ in the time covariate of the model by resetting the time at day 102 postinfection when drug treatment was stopped, since bacterial number had decreased significantly during the course of treatment. This turned out to be 6.59 days, which is consistent with the assumptions that the recall immune response is faster than the primary immune response or that the adaptive immune effect stayed at a relatively high level during treatment (16, 33), which results in much slower bacterial growth after treatment stops. With the reestimated *T*_50_ value indicating faster onset of immune effect, our baseline model was able to predict not only bacterial growth during the primary infection but also slower growth during relapse (Fig. 6).

### Significant impact of adaptive immune response on assessing drug effect.

**FIG 7:**
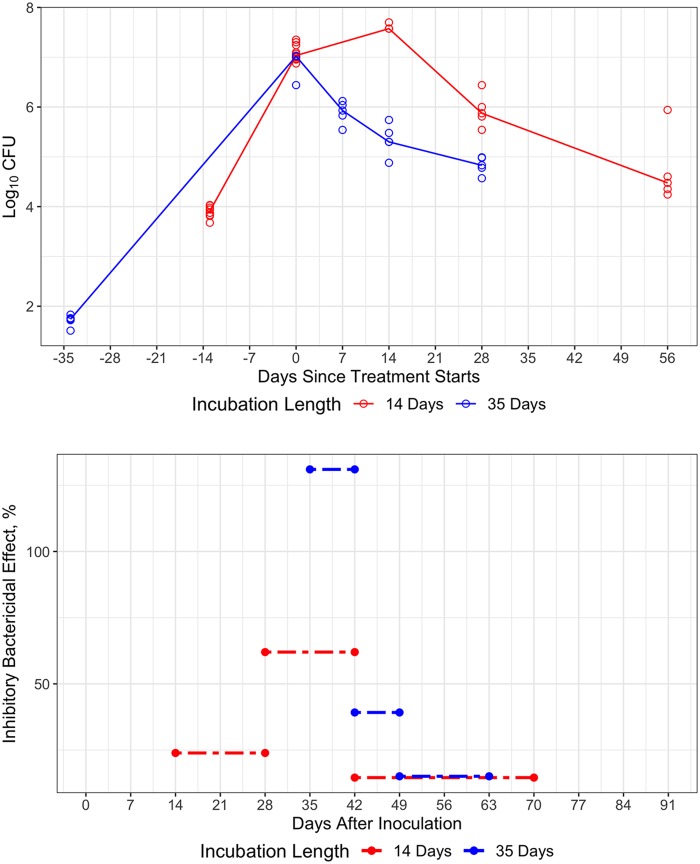
Differential treatment response of PZA monotherapy (top) and time-varying drug effect of PZA (bottom) in chronic (35 days) and subacute study (14 days) in BALB/c mice. Circle, raw data observed; solid line, median value of raw data; – - – -, predicted effect. Drug effect at a similar time period postinfection is similar between subacute and chronic infection study, although the incubation period without drug treatment is different.

We previously observed very different effects of pyrazinamide (PZA) in mouse efficacy studies, depending on the duration of the incubation period and the immune competence of the mouse strain (11, 14), leading us to consider that the PZA effect is dependent on the adaptive immune response (15, 34). Modeling these prior data using our baseline model, we observed that during treatment with PZA alone, the bacterial number followed very different time courses in the subacute model than those in the chronic model, despite starting at a similar level in both models (∼6.5 to 7.3 log_10_ CFU) (Fig. 7, top). In the subacute model, bacterial number initially increased by around one-half log_10_ CFU for the first 14 days of treatment (to ∼7.7 log_10_ CFU), and then decreased by around 1.5 log_10_ CFU and 3 log_10_ CFU (to ∼6 and ∼4.7 log_10_ CFU) on days 28 and 56, respectively, of treatment. In contrast, bacterial number decreased immediately with treatment in the chronic infection model, i.e., by 0.5 log_10_ CFU (to ∼6 log_10_ CFU) after 7 days of the same treatment in the chronic model, and by another ∼0.5 log_10_ CFU and 1 log_10_ CFU (to ∼5.5 log_10_ CFU and ∼5log_10_ CFU) on days 14 and 28, respectively, of treatment. Similarly, we estimated the drug effect of PZA as a percent inhibitory effect on bacterial growth on top of the adaptive immune effect for each period between sampling points, using our model with the assumption that the adaptive immune effect followed the sigmoidal function of bacterial number and time until it reached the highest level during the incubation period, which is around day 14 after treatment started for the subacute model and day 0 for the chronic model. It was found that the effect estimate of PZA was 23.9% inhibition for days 0 to 14, 62.0% inhibition for days 14 to 28, and 14.6% inhibition for days 28 to 56 of treatment (days 14–28, 28 to 42, and 42 to 70 postinfection, respectively) in the subacute infection model, compared to 131% inhibition for days 0 to 7 (an inhibitory effect greater than 100% implies that PZA kills the bacteria immediately when it is on board), 39.2% inhibition for days 7 to 14, and 15.1% inhibition for days 14 to 28 of treatment (days 35 to 42, 42 to 49, and 49 to 63 postinfection, respectively) in the chronic infection model (Fig. 7, bottom). This shows that drug effect of PZA differs at different stages during incubation and is likely significantly influenced by the development of the adaptive immune response such that, at a similar infection time, the PZA effect is comparable in the subacute and chronic models (Fig. 7, bottom).

### Protective immune effect in BALB/c mice as TB vaccine model.

**FIG 8:**
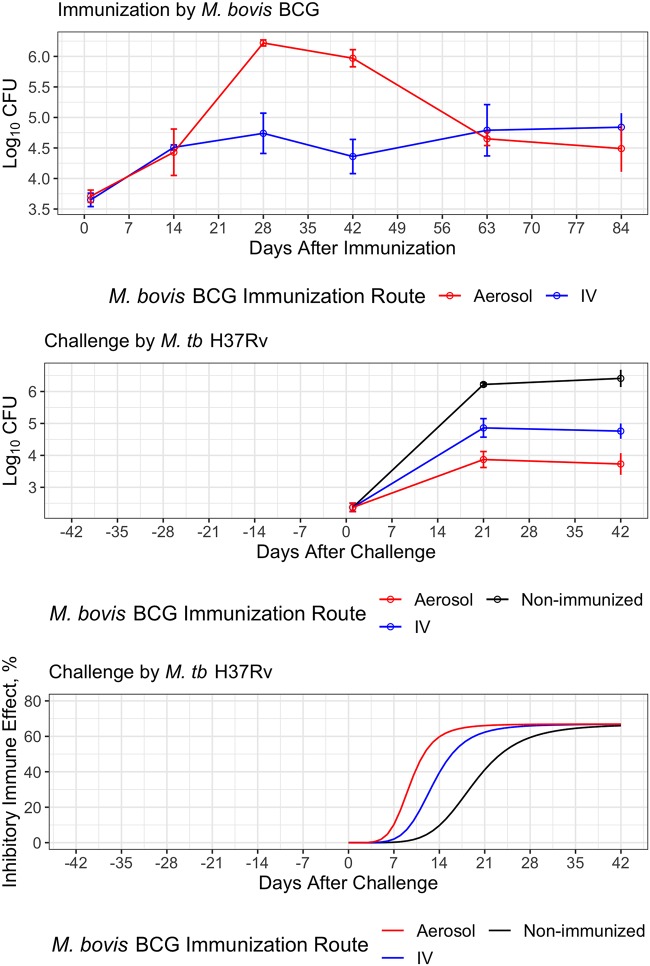
Bacterial growth and adaptive immune effect profiles in the immunization and challenge study. Circle, median value of raw data observed; solid line, profiles of the median value of raw data. (Top) M. bovis BCG growth profiles via aerosol or intravenous infection in the immunization study. (Middle) M. tuberculosis H37Rv growth profiles in mice in the challenge study immunized via aerosol or intravenous infection of M. bovis BCG and in naive mice. (Bottom) Simulated immune effect described by the baseline model with different *T*_50_ values for the infection of M. tuberculosis H37Rv in mice in the challenge study.

The baseline model that describes the bacterial growth with primary immune response and recall immune response was then tested using data from a paucibacillary mouse TB model following prior immunization with M. bovis BCG by the aerosol or intravenous route (35). Six weeks after immunization, mice were aerosol challenged with virulent Mycobacterium tuberculosis H37Rv (Fig. 8). Although the mice immunized via different routes started with almost identical counts of BCG in the lung on days 1 and 14 postimmunization, they ended up with significantly different CFU counts at the peak on day 28 and later reached a similar plateau on days 63 and 84 (Fig. 8, top). The growth profile of M. tuberculosis after challenge differed depending on whether the mice were immunized and on the route of immunization (Fig. 8, middle). Compared to nonimmunized mice, immunized mice had much lower CFU counts after challenge, with aerosol-immunized mice having the lowest counts. This is the inverse of the growth profiles of M. bovis BCG in the immunization period, where aerosol-immunized mice contained higher CFU counts, which resulted in a greater adaptive immune response according to our model. The immune effect acquired in the immunized and nonimmunized mice via aerosol challenge of M. tuberculosis H37Rv was reestimated by assuming that the dynamics of the protective cell-mediated memory response to aerosol challenge is accelerated like that observed after the end of drug treatment in the relapse study. Similarly, we reestimated *T*_50_ in the time covariate of the model, which turned out to be 13.1 days for mice immunized via the intravenous route and 9.54 days for mice immunized via the aerosol route, compared to 19.3 days for nonimmunized mice (Fig. 8, bottom). Reestimation of other parameters, including *K_T_*, *K_B_*, *B*_50_, *K_T_*, and *T*_50_, or *K_B_* and *B*_50_, did not result in altered estimates and/or better model fitting. This result also supports the assumption that the protective memory response acts faster in the BCG-primed mice than in the naive mice and when immunization occurs via the aerosol route compared to the intravenous route, consistent with the ability of antigen-specific memory T cells to get to the lungs quickly upon infection and/or to more rapidly populate the lung as a measure of the recall adaptive immune response.

## DISCUSSION

The differences in bacterial growth profiles between BALB/c and athymic nude mice observed in the data that we collected are intuitive (Fig. 1 and Fig. S1 and S2). The interplay of infectious dose, the timing of the onset and the magnitude of the adaptive immune effect, and the incubation time is critical regarding the bacterial infection dynamics and the survival of mice. Within the same incubation time, BALB/c mice receiving low infectious doses were less likely to die than those receiving high infectious doses and athymic nude mice receiving low or high inoculums. All of these factors, including bacterial number, incubation time, and maximal CFU- and time-dependent adaptive immune effects, have been included in the model to describe the adaptive immune response during M. tuberculosis infection of mice (equation 3).

Our model shows, for the first time, that the adaptive immune effect in mouse TB models is dependent on both bacterial number and duration of incubation (Fig. 5). The heat map plot shows the 3-dimensional relationship between bacterial number, incubation time, and total adaptive immune effect for BALB/c and athymic nude mice (Fig. 5, top right and bottom right). The time to the onset of the adaptive immune effect is around 7 to 9 days after infection for BALB/c mice (transition from blue to gray shaded ribbon area) and it takes 25 to 28 days to reach the maximal level (dark red shaded ribbon area), which is consistent with prior observations from animal studies (6). The levels of infectious dose do not affect the magnitude of the adaptive immune effect significantly, since the color change over the incubation time is almost the same across the infectious dose range in the shaded ribbon area. Based on our model, a 10-fold infectious dose would only increase the maximal immune effect by no more than 5% inhibition on the growth on day 28 for the four arms receiving different infectious doses in BALB/c mice (Fig. 5, top right, and Fig. S2). This implies that the arrival of the plateau of the adaptive immune effect can only be accelerated by 1 or 2 days (16). For athymic nude mice with a modest amount of T-cell maturation outside the thymus, the immune effect starts around the same time as in BALB/c mice, but the magnitude of both the maximal CFU- and time-dependent immune effects is much lower than in BALB/c mice, as expected (Fig. 5, bottom right, and Table 1). Interestingly, the bacterial number at which the half-maximal CFU-dependent immune effect appears is much lower for nude mice, with a slightly longer time to reach the half-maximal time covariate than BALB/c mice (Fig. 5, top left and bottom left, and Table 1).

Precise quantification of bacterial growth dynamics and immune response was necessary to accurately describe the efficacy of pyrazinamide in subacute and chronic mouse models. PZA is a key sterilizing drug in the first-line TB regimen. Its antibacterial activity is highly dependent on the pH of the environment *in vitro* and *in vivo*, with MICs decreasing by an order of magnitude with each unit change in pH values (37). PZA exerts strong initial bactericidal activity in the chronic BALB/c mouse infection model, since the infecting bacilli are virtually all intracellular, residing in the acidified environment in the phagosomes of activated macrophages, where the pH can be as low as 4.5 (34, 37). On the other hand, the bactericidal effect of PZA monotherapy in the subacute BALB/c infection model is delayed. The slight increase in CFU counts during the first 2 weeks of treatment in this model, before the peak of the adaptive immune response, is presumably due to the lack of a suitably acidic environment in naive macrophages for the active metabolite of PZA, pyrazinoic acid (POA), to become protonated and to permeate through the membrane into the bacilli (38). Since the adaptive immune response takes around 17 days to reach half of the maximal time covariate, which accounts for 70% inhibition in our model, it implies that a longer incubation time is required before the phagosomes containing the bacilli mature and acidify following activation by T cells (5, 6). This explains why, after 14 days of treatment in the subacute study (to reach day 28 postinfection), PZA started exerting bactericidal activity similar to that observed in the chronic infection model in which treatment was initiated 35 days postinfection (Fig. 7). In our baseline model, it was found that the adaptive immune effect reaches its maximum level after 25 to 28 days of incubation without any treatment in BALB/c mice (Fig. 5, top right, dark red shaded ribbon area). Therefore, a precise description of the development of adaptive immune effects in various mouse models plays an important role in understanding the dynamics of PZA activity (11, 15) and should be similarly useful for understanding the activity of other drugs that may depend on a component of the immune response for optimal activity.

The relatively low CFU counts that bacteria reached in relapsing mice implied that the adaptive immune effect stimulated by primary infection might either remain high or gradually decline during treatment, as the bacterial number decreases to a much lower level before the regrowth (Fig. 6). We considered that the bacteria that regrow arise from persistent or resistant bacteria with mutations selected by the chemotherapy, which might grow more slowly due to a mutation-induced loss of fitness. However, experience tells us that the relapsing bacteria are fully drug susceptible and equally fit as the wild type, at least in most cases, so it is challenging to accept that the bacteria causing relapse are somehow less fit. Instead, an immunizing effect of the primary infection that limits the regrowth of bacteria in the lung during relapse fits better here since it is similar to the scenario of vaccination, in which adaptive immunity against the infection has been acquired. In fact, the vaccination/challenge study supported this explanation, in that a similar plateau around 4 log_10_ CFU was observed in the mice which were immunized with a BCG vaccine strain via the aerosol route and then infected with M. tuberculosis via aerosol 6 weeks later as a challenge (Fig. 8) (35). In contrast, in the mice without prior immunization with BCG, bacterial growth followed the typical profile up to 6 log_10_ CFU or more (Fig. 1). In the relapse study, although it is not entirely clear whether the adaptive immune effect decreased during the treatment period or remained at the same high level as that when treatment started, a shorter *T*_50_ indicating faster immune response onset (almost instantaneous) was used to describe the lower plateau of CFU counts during relapse. This suggests that either the recall adaptive immunity was reactivated to immediately reach a high level or that it never faded away. While the fact that the bacterial counts increased at all strongly suggests that the immune effect must have decreased to some degree during treatment, more intensive sampling of CFU counts during the early growth period of reinfection or relapse is needed to further investigate this detail. In the case of challenge infection after immunization, the ability of memory T cells either to populate the lung or to get to the lungs quickly upon infection determines the recall protective adaptive immune response (16). It is also interesting to see that the reestimated *T*_50_ may be inversely related to the plateau level of M. bovis BCG in the immunization period, which implies the quantity of the memory T cells could determine the recall response (Fig. 8). Therefore, our baseline model can also be used for characterizing the dose-response relationship of TB vaccines. Besides the application of our model in reinfection/relapse, some considerations about modeling exposure-response relationships of TB drugs or regimens have been explored, such as how to formulate the adaptive immune effect when drug is on board. The adaptive immune effect may never go away or could be rapidly recalled, even if bacterial numbers drop significantly. According to our model, the time-dependent component contributes much more than the CFU-dependent component to the magnitude of the immune effect. Therefore, the drop in bacterial number may not make as significant an impact on the level of immune effect. This assumption regarding the change of adaptive immune effect during treatment period will be further investigated experimentally and mathematically.

In conclusion, we present here a mechanism-based baseline model of M. tuberculosis infection in BALB/c and athymic nude mice, which mathematically describes the dynamics of bacterial growth and the adaptive immune effect. We also expanded this model to describe the bacterial profile during treatment with PZA, during relapse after inadequate treatment, and during challenge infection after vaccination. As pointed out, the adaptive immune response has a deterministic role in the evolution of infecting bacterial populations throughout the period of infection, including assisting the action of PZA, as well as mediating protective effects via prior infection and vaccination. Our baseline model is therefore a useful tool to quantify the adaptive immune effect elicited by pathogenic mycobacteria or preventive vaccines. The quantitation of the adaptive immune effect will be important to understand the efficacy of TB drugs, regimens, and vaccines in preclinical studies and provide better prediction of clinical trial outcomes for new treatment against TB disease.

## MATERIALS AND METHODS

### Experimental data from studies evaluating growth in untreated mice.

In total, data from 74 and 12 published and unpublished mouse efficacy studies evaluating TB drugs or regimens in BALB/c and athymic nude mice, respectively, were collected. Mice in each experiment were block randomized to treatment assignment after aerosol infection of M. tuberculosis H37Rv. The infectious dose ranged from 0.5 to 4.8 log_10_ CFU, depending on the experiment. Lung CFU counts were collected at prescribed time points to assess the growth of M. tuberculosis.

An additional study was performed to supplement the historical data by evaluating the impact of infectious dose size on bacterial growth in BALB/c and athymic nude mice. The infectious doses evaluated included below 2 log_10_ CFU, 2 to 3 log_10_ CFU, 3 to 4 log_10_ CFU, and more than 4 log_10_ CFU. Mice of each strain were block randomized to different infectious doses of M. tuberculosis H37Rv administered via aerosol. Three mice per strain were sacrificed and lung CFU counts were collected at days 1, 2, 4, 7, 11, 14, 16, 18, 21, 23, 25, 28, 30, 32, 35, 42, 46, 49, and 50 postinfection to assess the growth of M. tuberculosis.

### Experimental data from a study evaluating relapse after combination chemotherapy.

Mice were block randomized to treatment groups after low-dose aerosol infection of M. tuberculosis H37Rv and incubated for 32 days before treatment with a combination of rifampin (10 mg/kg), isoniazid (10 mg/kg), and PZA (150 mg/kg) (RHZ) dosed daily by gavage, 5 days per week for 10 weeks. After the treatment stopped, the mice were kept for another 6 weeks to capture the regrowth. Lung CFU counts were collected at prescribed time points during and after treatment to assess the growth of M. tuberculosis.

### Experimental data from studies evaluating response to PZA treatment.

For the studies that evaluated efficacy of PZA monotherapy, mice in each experiment were block randomized to treatment groups after low-dose aerosol infection of M. tuberculosis H37Rv and incubated for 14 days (subacute model) or 35 days (chronic model) before treatment with PZA. PZA (150 mg/kg) was dosed daily by gavage, 5 days per week, for 8 weeks (subacute model) and 4 weeks (chronic model). Lung CFU counts were collected at prescribed time points to assess the growth of M. tuberculosis.

### Experimental data from a study evaluating the effect of BCG immunization on M. tuberculosis challenge infection.

Mice were block randomized to receive either aerosol immunization or intravenous immunization with M. bovis BCG or to a control arm without immunization, as previously described (35). Six weeks after immunization, all mice were challenged by the aerosol route with M. tuberculosis H37Rv. CFU counts were collected at day 1, week 2, week 4, and week 6 postimmunization to assess the growth of BCG and at day 1, week 3, and week 6 postchallenge to assess the growth of BCG and M. tuberculosis after challenge.

### Model development.

Model development and data analysis was conducted using nonlinear mixed-effects modeling (NONMEM 7 software; ICON Development Solutions, San Antonio, TX) (39). Data analyses, diagnostics, and visualization were performed using R packages (R-3.1.1, Development Core Team) (40). The first-order conditional estimation with interaction method (FOCEI) was used.

Bacterial growth was described by a baseline model, which accounts for bacterial replication, bacterial natural death, and inhibition imposed by the adaptive immune response. The adaptive immune effect was described by a sigmoidal function depending on the scenarios described. The recall adaptive immune effect in the relapse study and the immunization/challenge study was formulated the same as that in the baseline model, with one or two parameters reestimated. Model optimization was guided by the likelihood ratio test (LRT) for the nested model, diagnostic plots, and internal model validation techniques, including visual predictive checks. When additional parameters were included, a decrease of 3.84 points (*P* < 0.05 for 1 degree of freedom [df]) was considered statistically different.

The combined effect of RHZ treatment in the relapse study and the effects of PZA treatment in the monotherapy studies were estimated as constant values during each period between sampling time points. Observed drug effects were estimated as percent changes on top of the adaptive immune effect on the bacterial growth rate in the baseline model.

Visual diagnostic plots at all major steps of model development were used to characterize goodness of fit of observations to predictions for both BALB/c and athymic nude mice. VPCs were performed to evaluate the simulation properties of the final baseline models, using 200 simulations based on final baseline parameter values (including the inter- and intraindividual variability) for both BALB/c and athymic nude mouse models.

### Data availability.

The data used in this research were generated in-house. The data in this work are mainly the CFU counts over incubation time in a TB mouse model with or without any chemotherapy treatment. Data are available upon request.

## Supplementary Material

Supplemental file 1

## References

[1] World Health Organization. 2018 Global tuberculosis report. World Health Organization, Geneva, Switzerland.

[2] Kwon BE, Ahn JH, Min S, Kim H, Seo J, Yeo SG, Ko HJ 2018 Development of new preventive and therapeutic vaccines for tuberculosis. Immune Netw 18:e17. doi:10.4110/in.2018.18.e17. 29732235 PMC5928416

[3] Tiberi S, Du Plessis N, Walzl G, Vjecha MJ, Rao M, Ntoumi F, Mfinanga S, Kapata N, Mwaba P, McHugh TD, Ippolito G, Migliori GB, Maeurer MJ, Zumla A 2018 Tuberculosis: progress and advances in development of new drugs, treatment regimens, and host-directed therapies. Lancet Infect Dis 18:e183–e198. doi:10.1016/S1473-3099(18)30110-5. 29580819

[4] Dartois V 2014 The path of anti-tuberculosis drugs: from blood to lesions to mycobacterial cells. Nat Rev Microbiol 12:159–167. doi:10.1038/nrmicro3200. 24487820 PMC4341982

[5] Bozzano F, Marras F, De Maria A 2014 Immunology of tuberculosis. Mediterr J Hematol Infect Dis 6:e2014027. doi:10.4084/MJHID.2014.027. 24804000 PMC4010607

[6] Ernst JD 2012 The immunological life cycle of tuberculosis. Nat Rev Immunol 12:581–591. doi:10.1038/nri3259. 22790178

[7] Moliva JI, Turner J, Torrelles JB 2017 Immune responses to bacillus Calmette-Guérin vaccination: why do they fail to protect against *Mycobacterium tuberculosis*? Front Immunol 8:407–407. doi:10.3389/fimmu.2017.00407. 28424703 PMC5380737

[8] Zhang M, Li SY, Rosenthal IM, Almeida DV, Ahmad Z, Converse PJ, Peloquin CA, Nuermberger EL, Grosset JH 2011 Treatment of tuberculosis with rifamycin-containing regimens in immune-deficient mice. Am J Respir Crit Care Med 183:1254–1261. doi:10.1164/rccm.201012-1949OC. 21330452 PMC3114054

[9] Park SW, Tasneen R, Converse PJ, Nuermberger EL 2017 Immunodeficiency and intermittent dosing promote acquired rifamycin monoresistance in murine tuberculosis. Antimicrob Agents Chemother 61:e01502-17. doi:10.1128/AAC.01502-17. 28874368 PMC5655103

[10] Xu J, Li S-Y, Almeida DV, Tasneen R, Barnes-Boyle K, Converse PJ, Upton AM, Mdluli K, Fotouhi N, Nuermberger EL, Xu J, Li S-Y, Almeida DV, Tasneen R, Barnes-Boyle K, Converse PJ, Upton AM, Mdluli K, Fotouhi N, Nuermberger EL 2019 Contribution of pretomanid to novel regimens containing bedaquiline with either linezolid or moxifloxacin and pyrazinamide in murine models of tuberculosis. Antimicrob Agents Chemother 63:e00021-19. doi:10.1128/AAC.00021-19. 30833432 PMC6496099

[11] Almeida DV, Tyagi S, Li S, Wallengren K, Pym AS, Ammerman NC, Bishai WR, Grosset JH 2014 Revisiting anti-tuberculosis activity of pyrazinamide in mice. Mycobact Dis 4:145. doi:10.4172/2161-1068.1000145. 25525563 PMC4267256

[12] Zhang Y, Mitchison D 2003 The curious characteristics of pyrazinamide: a review. Int J Tuber Lung Dis 7:6–21. 12701830

[13] Zhan L, Tang J, Sun M, Qin C 2017 Animal models for tuberculosis in translational and precision medicine. Front Microbiol 8:717. doi:10.3389/fmicb.2017.00717. 28522990 PMC5415616

[14] Nuermberger E 2011 The role of the mouse model in the evaluation of new antituberculosis drugs, p 145–152. *In* Donald PR, van Helden PD (ed), Antituberculosis chemotherapy. Karger AG, Basel, Switzerland.

[15] Nuermberger EL 2017 Preclinical efficacy testing of new drug candidates. Microbiol Spectr 5. doi:10.1128/microbiolspec.TBTB2-0034-2017. PMC11687513 28643624

[16] Cooper AM 2009 Cell-mediated immune responses in tuberculosis. Annu Rev Immunol 27:393–422. doi:10.1146/annurev.immunol.021908.132703. 19302046 PMC4298253

[17] Franzblau SG, DeGroote MA, Cho SH, Andries K, Nuermberger E, Orme IM, Mdluli K, Angulo-Barturen I, Dick T, Dartois V, Lenaerts AJ 2012 Comprehensive analysis of methods used for the evaluation of compounds against *Mycobacterium tuberculosis*. Tuberculosis (Edinb) 92:453–488. doi:10.1016/j.tube.2012.07.003. 22940006

[18] Eftimie R, Gillard JJ, Cantrell DA 2016 Mathematical models for immunology: current state of the art and future research directions. Bull Math Biol 78:2091–2134. doi:10.1007/s11538-016-0214-9. 27714570 PMC5069344

[19] Kirschner D, Pienaar E, Marino S, Linderman JJ 2017 A review of computational and mathematical modeling contributions to our understanding of *Mycobacterium tuberculosis* within-host infection and treatment. Curr Opin Syst Biol 3:170–185. doi:10.1016/j.coisb.2017.05.014. 30714019 PMC6354243

[20] Pienaar E, Cilfone NA, Lin PL, Dartois V, Mattila JT, Butler JR, Flynn JL, Kirschner DE, Linderman JJ 2015 A computational tool integrating host immunity with antibiotic dynamics to study tuberculosis treatment. J Theor Biol 367:166–179. doi:10.1016/j.jtbi.2014.11.021. 25497475 PMC4332617

[21] Cilfone NA, Perry CR, Kirschner DE, Linderman JJ 2013 Multi-scale modeling predicts a balance of tumor necrosis factor-α and interleukin-10 controls the granuloma environment during *Mycobacterium tuberculosis* infection. PLoS One 8:e68680. doi:10.1371/journal.pone.0068680. 23869227 PMC3711807

[22] Pitcher MJ, Bowness R, Dobson S, Gillespie SH 2018 A spatially heterogeneous network-based metapopulation software model applied to the simulation of a pulmonary tuberculosis infection. Appl Netw Sci 3:33. doi:10.1007/s41109-018-0091-2. 30839831 PMC6214320

[23] Marino S, El-Kebir M, Kirschner D 2011 A hybrid multi-compartment model of granuloma formation and T cell priming in tuberculosis. J Theor Biol 280:50–62. doi:10.1016/j.jtbi.2011.03.022. 21443879 PMC3740747

[24] Ganguli S, Gammack D, Kirschner DE 2005 A metapopulation model of granuloma formation in the lung during infection with *Mycobacterium tuberculosis*. Math Biosci Eng 2:535–560. doi:10.3934/mbe.2005.2.535. 20369939

[25] Fallahi-Sichani M, El-Kebir M, Marino S, Kirschner DE, Linderman JJ 2011 Multiscale computational modeling reveals a critical role for TNF-α receptor 1 dynamics in tuberculosis granuloma formation. Ji 186:3472–3483. doi:10.4049/jimmunol.1003299. PMC3127549 21321109

[26] Bartelink IH, Zhang N, Keizer RJ, Strydom N, Converse PJ, Dooley KE, Nuermberger EL, Savic RM 2017 New paradigm for translational modeling to predict long-term tuberculosis treatment response. Clin Transl Sci 10:366–379. doi:10.1111/cts.12472. 28561946 PMC5593171

[27] Kennedy JD, Pierce CW, Lake JP 1992 Extrathymic T cell maturation. Phenotypic analysis of T cell subsets in nude mice as a function of age. J Immunol 148:1620–1629. 1347303

[28] Nielsen EI, Friberg LE 2013 Pharmacokinetic-pharmacodynamic modeling of antibacterial drugs. Pharmacol Rev 65:1053–1090. doi:10.1124/pr.111.005769. 23803529

[29] Gill WP, Harik NS, Whiddon MR, Liao RP, Mittler JE, Sherman DR 2009 A replication clock for *Mycobacterium tuberculosis*. Nat Med 15:211–214. doi:10.1038/nm.1915. 19182798 PMC2779834

[30] Drusano GL, Fregeau C, Liu W, Brown DL, Louie A 2010 Impact of burden on granulocyte clearance of bacteria in a mouse thigh infection model. Antimicrob Agents Chemother 54:4368–4372. doi:10.1128/AAC.00133-10. 20516275 PMC2944594

[31] Drusano GL, Liu W, Kulawy R, Louie A 2011 Impact of granulocytes on the antimicrobial effect of tedizolid in a mouse thigh infection model. Antimicrob Agents Chemother 55:5300–5305. doi:10.1128/AAC.00502-11. 21911576 PMC3195040

[32] Drusano GL, Vanscoy B, Liu W, Fikes S, Brown D, Louie A 2011 Saturability of granulocyte kill of *Pseudomonas aeruginosa* in a murine model of pneumonia. Antimicrob Agents Chemother 55:2693–2695. doi:10.1128/AAC.01687-10. 21422203 PMC3101416

[33] Kirman JR, Henao-Tamayo MI, Agger EM 2016 The memory immune response to tuberculosis. Microbiol Spectr 4. doi:10.1128/microbiolspec.TBTB2-0009-2016. 28087940

[34] Lanoix JP, Ioerger T, Ormond A, Kaya F, Sacchettini J, Dartois V, Nuermberger E 2016 Selective inactivity of pyrazinamide against tuberculosis in C3HeB/FeJ mice is best explained by neutral pH of caseum. Antimicrob Agents Chemother 60:735–743. doi:10.1128/AAC.01370-15. 26574016 PMC4750710

[35] Nuermberger EL, Yoshimatsu T, Tyagi S, Bishai WR, Grosset JH 2004 Paucibacillary tuberculosis in mice after prior aerosol immunization with *Mycobacterium bovis* BCG. Infect Immun 72:1065–1071. doi:10.1128/iai.72.2.1065-1071.2004. 14742554 PMC321637

[36] Reference deleted.

[37] Zhang Y, Permar S, Sun Z 2002 Conditions that may affect the results of susceptibility testing of *Mycobacterium tuberculosis* to pyrazinamide. J Med Microbiol 51:42–49. doi:10.1099/0022-1317-51-1-42. 11800471

[38] Zhang Y, Shi W, Zhang W, Mitchison D 2013 Mechanisms of pyrazinamide action and resistance. Microbiol Spectr 2:1–12. doi:10.1128/microbiolspec.MGM2-0023-2013. 25530919 PMC4268777

[39] Beal SL, Sheiner LB, Boeckmann AJ, Bauer RJ 2019 NONMEM user’s guides (1989–2019). Icon Development Solutions, Ellicott City, MD.

[40] Keizer RJ, Karlsson MO, Hooker A 2013 Modeling and simulation workbench for NONMEM: tutorial on Pirana, PsN, and Xpose. CPT Pharmacometrics Syst Pharmacol 2:e50. doi:10.1038/psp.2013.24. 23836189 PMC3697037

