# Supplementary material for: Mechanistic Modeling of Mycobacterium tuberculosis Infection in Murine Models for Drug and Vaccine Efficacy Studies
Source: Antimicrob Agents Chemother. 2020 Feb 21;64(3):e01727-19. doi: 10.1128/AAC.01727-19 (PMC7038312; doi:10.1128/AAC.01727-19)
Supplement: Supplemental file 1 [file AAC.01727-19-s0001.pdf]

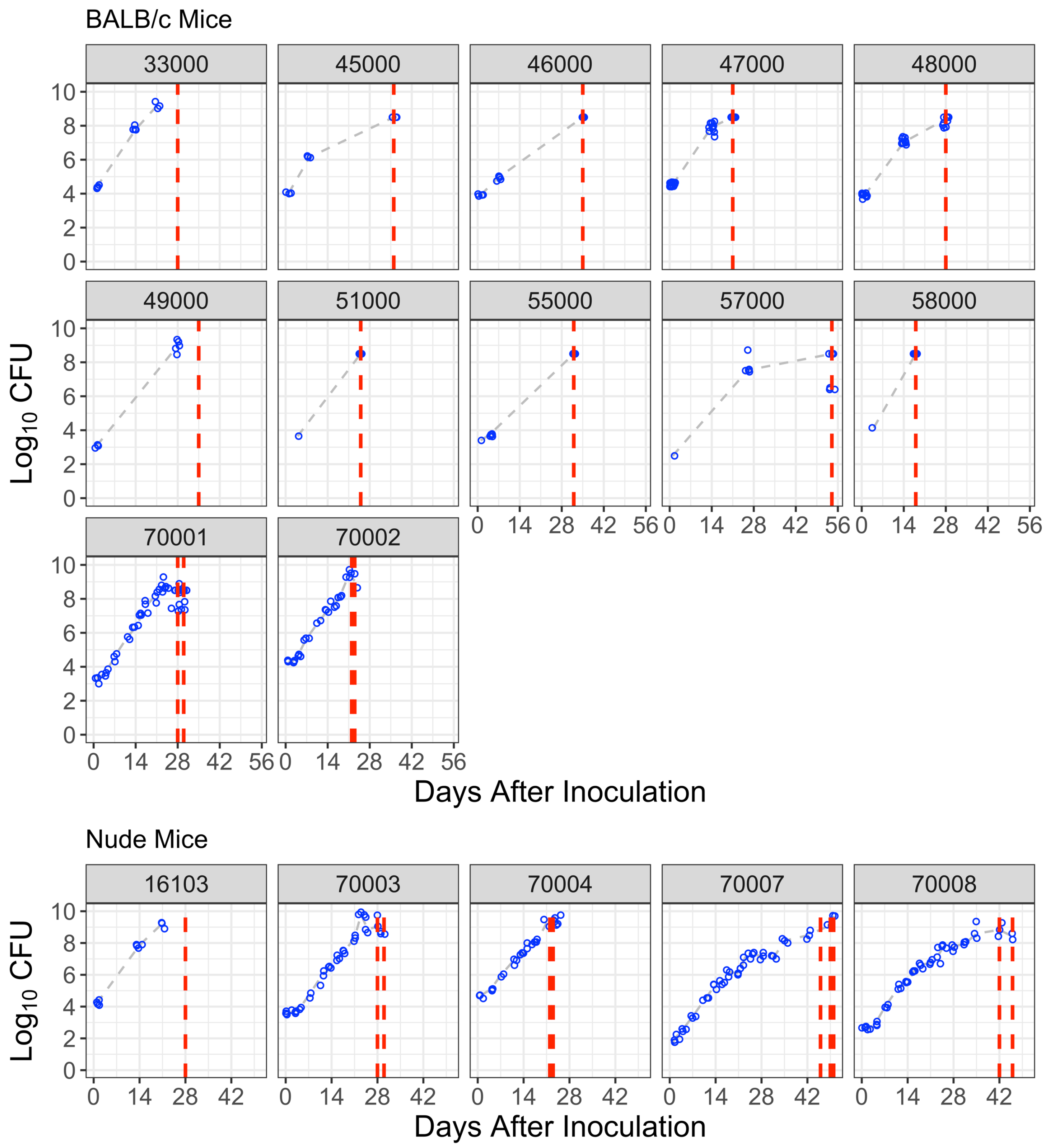


**Figure S1. Bacterial Growth Profiles with Death Event Records in BALB/c and Athymic Nude Mice.** The red dash line indicates the occurrence of the death events in mice.

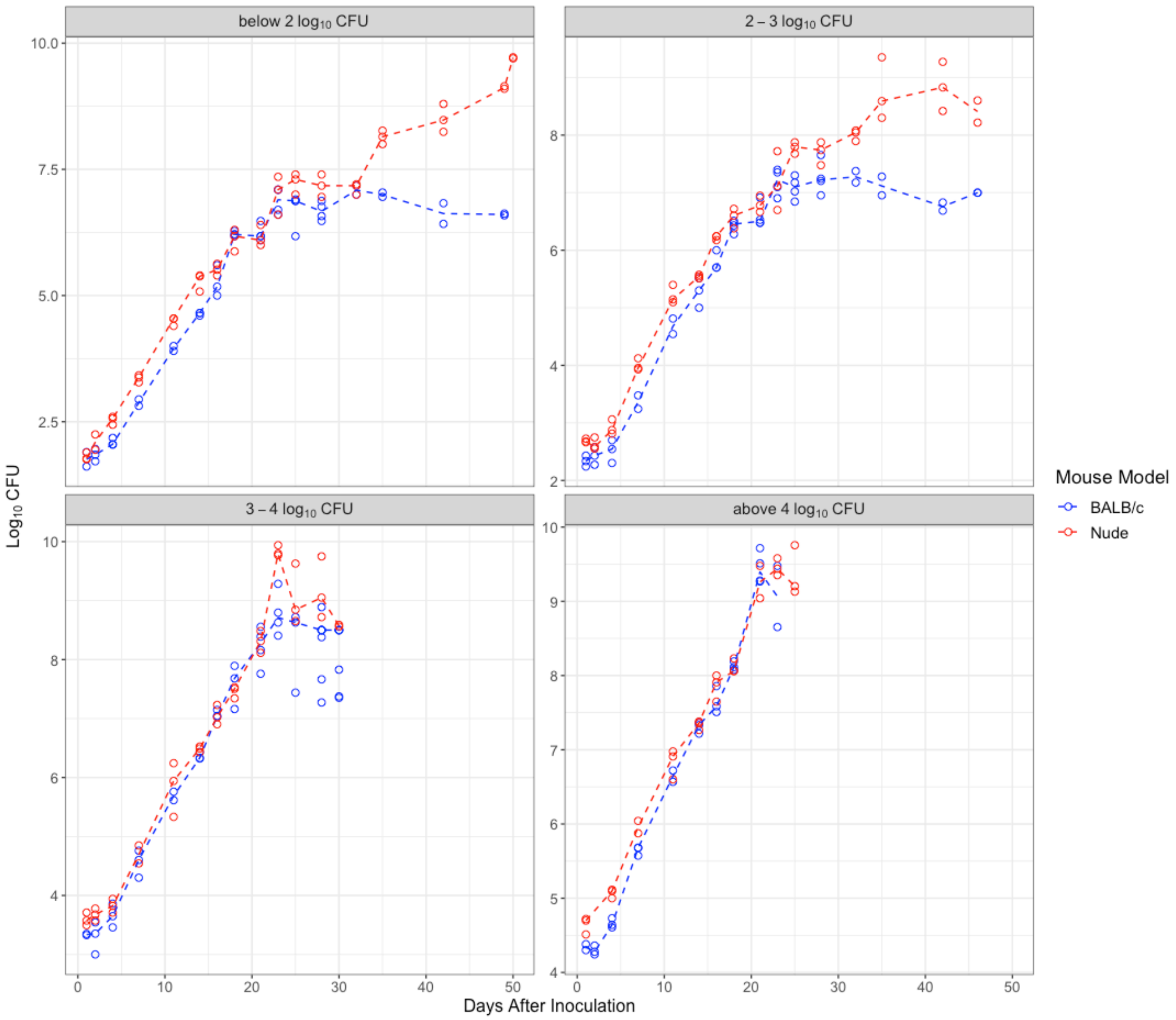


**Figure S2. Bacterial Growth Profiles with Different Inoculum in BALB/c (blue) and Athymic Nude Mice (red)**

## A) BALB/c Mice

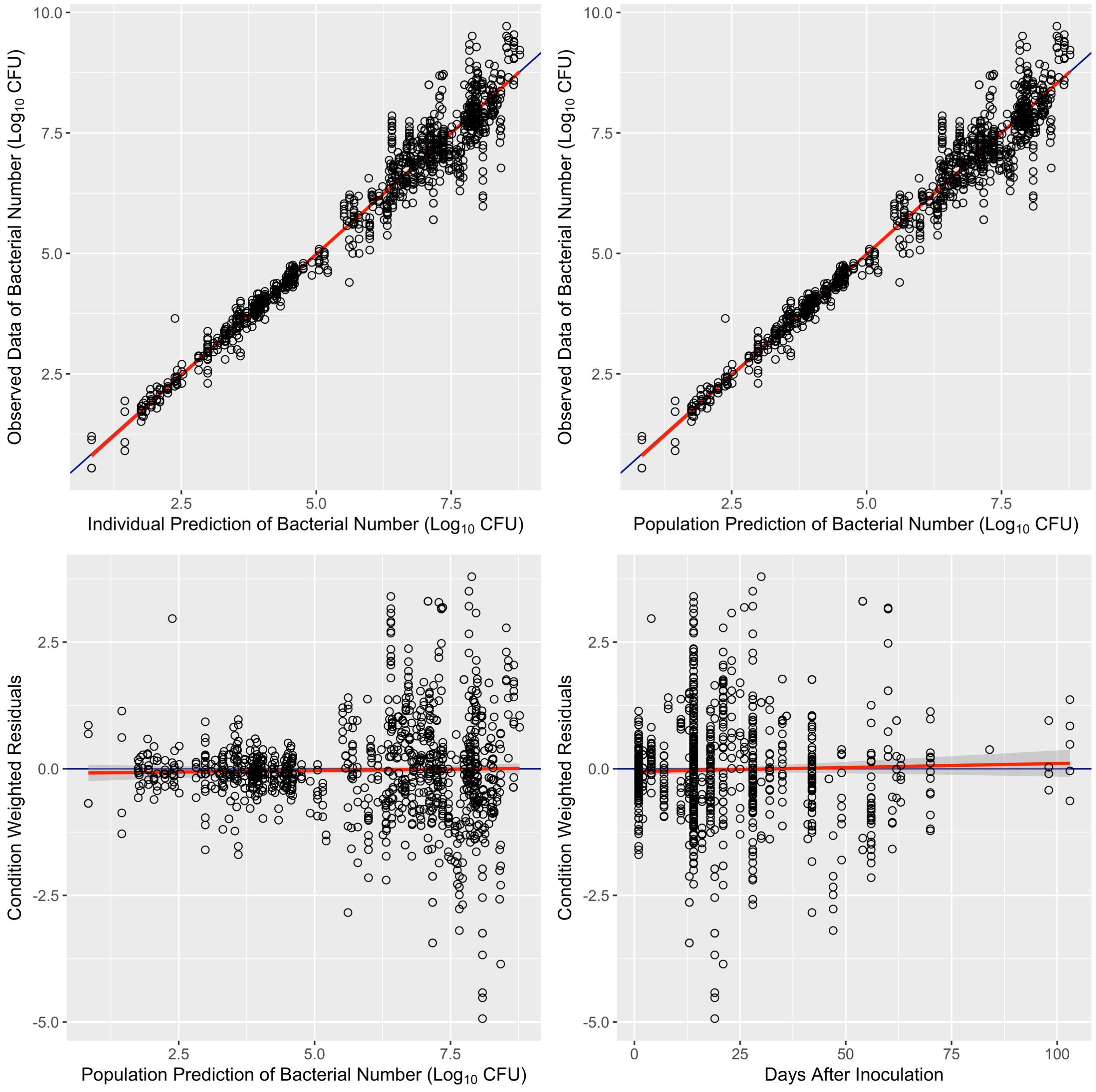
Observed Data of Bacterial Number (Log10 CFU)
Individual Prediction of Bacterial Number (Log10 CFU)
Population Prediction of Bacterial Number (Log10 CFU)
Condition Weighted Residuals
Days After Inoculation
10.0
7.5
5.0
2.5
0.0
-2.5
-5.0
0
25
50
75
100

## B) Nude Mice

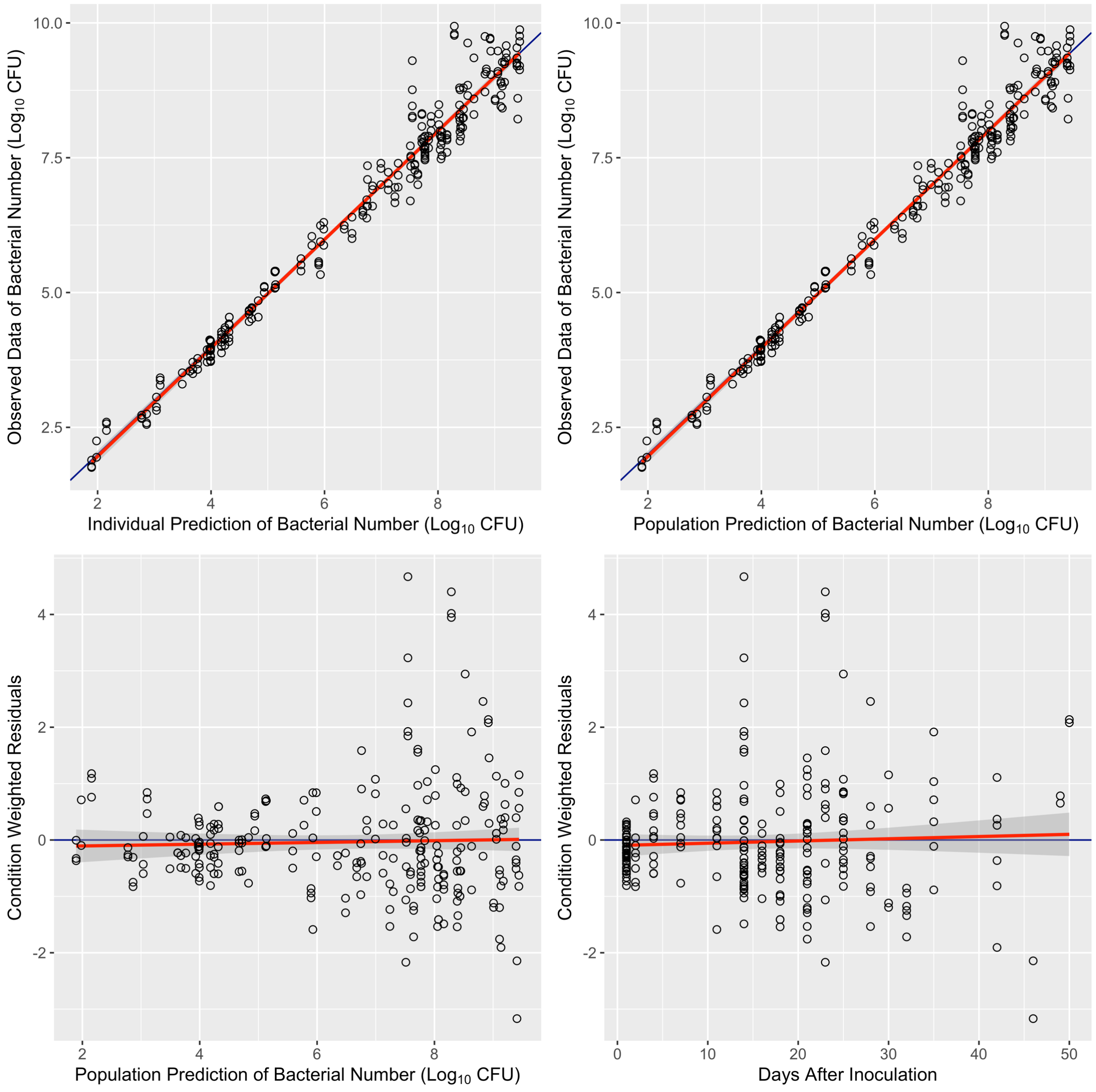


**Figure S3. Goodness of Fit Plot of the Baseline Model for BALB/c (A) and Athymic Nude (B) Mice**
